# Safety relevant knowledge of orally anticoagulated patients without self-monitoring: a baseline survey in primary care

**DOI:** 10.1186/1471-2296-15-104

**Published:** 2014-05-25

**Authors:** Jean-François Chenot, Thanh Duc Hua, Manar Abu Abed, Hannelore Schneider-Rudt, Tim Friede, Simon Schneider, Stefan Viktor Vormfelde

**Affiliations:** 1 Department of General Practice, Institute for Community Medicine, University Medicine Greifswald, Greifswald, Germany; 2 Department of General Practice, University Medical Center Göttingen, Göttingen, Germany; 3 Department of Medical Statistics, University Medical Center Göttingen, Göttingen, Germany; 4 Institute of Clinical Pharmacology, University Medical Center Göttingen, Robert-Koch-Str. 40, D-37075 Göttingen, Germany

## Abstract

**Background:**

Effective and safe management of oral anticoagulant treatment (OAT) requires a high level of patient knowledge and adherence. The aim of this study was to assess patient knowledge about OAT and factors associated with patient knowledge.

**Methods:**

This is a baseline survey of a cluster-randomized controlled trial in 22 general practices with an educational intervention for patients or their caregivers. We assessed knowledge about general information on OAT and key facts regarding nutrition, drug-interactions and other safety precautions of 345 patients at baseline.

**Results:**

Participants rated their knowledge about OAT as excellent to good (56%), moderate (36%) or poor (8%). However, there was a discrepancy between self-rated knowledge and evaluated actual knowledge and we observed serious knowledge gaps. Half of the participants (49%) were unaware of dietary recommendations. The majority (80%) did not know which non-prescription analgesic is the safest and 73% indicated they would not inform pharmacists about OAT. Many participants (35-75%) would not recognize important emergency situations. After adjustment in a multivariate analysis, older age and less than 10 years education remained significantly associated with lower overall score, but not with self-rated knowledge.

**Conclusions:**

Patients have relevant knowledge gaps, potentially affecting safe and effective OAT. There is a need to assess patient knowledge and for structured education programs.

**Trial registration:**

Deutsches Register Klinischer Studien (German Clinical Trials Register): DRKS00000586.

Universal Trial Number (UTN U1111-1118-3464).

## Background

Thrombosis and atrial fibrillation are the major indications for oral anticoagulation therapy (OAT). Due to an increase in aging population, the number of adults with atrial fibrillation will increase considerably. Consequently, a global increase in the number of patients requiring OAT is expected over the next decades [1]. The effectiveness of OAT to reduce risk of stroke or recurrent thrombosis has been proven and OAT is recommended in guidelines [2,3]. Frequently used drugs for OAT are warfarin, phenprocoumon and acenocoumarol. Studies have shown that insufficient adherence and a low level of patient knowledge about OAT are the primary causes for complications [4-6]. OAT thus requires regular monitoring of the international normalized ratio (INR) and dose adjustments, as well as a high level of patient knowledge and adherence to recommendations on nutrition, medication and recognition of critical situations. Bleeding complications occur in 0.3 to 0.4% of all patients with OAT every year, but risk estimates vary widely [7,8]. While educational programs, self-monitoring and self-management of OAT have been established this is not suitable for many patients [9]. Standardized education programs for patients without self-monitoring are lacking in many countries [4,6]. The best strategy to educate patients about oral anticoagulation has not been determined yet [10,11]. In Germany, general practitioners manage the majority of patients with OAT, there are no coagulation clinics. Some general practitioners use self-written patient information brochures to inform patients about OAT. Content and quality of these brochures is heterogeneous and sometimes inaccurate. It is known that information brochures on OAT require reading levels, exceeding the capacity of many patients [12].

The aim of this study was to assess patients’ knowledge about OAT and factors associated with patient knowledge.

## Methods

This is a baseline survey within a cluster-randomized controlled study conducted in 22 general practices in Germany. The Ethics Committee of the medical school of the University of Göttingen approved this study. A detailed study protocol has been published [13].

### Recruitment of practices and patients

**Figure 1:**
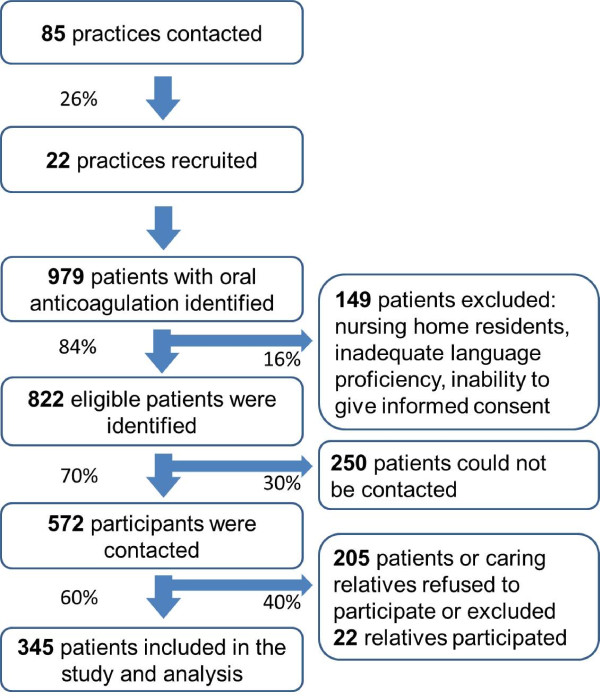
Recruitment flow.

A total of 22 general practices out of 85 practices agreed to participate in the study (2 practices did not exist anymore, 3 were put on a waiting list). Addresses were obtained from the local Association of Statutory Health Insurance Physicians (Kassenärztliche Vereinigung). The GPs (n = 32) were on average 12.7 years in practice (SD ± 6.9), 49 years old (SD ± 6) (national average 50.4 years) and 52% of them were female (national average 38%). A total of 18 (80%) practices were run by a single GP. The study was conducted in two medium size university cities (5 practices) and surrounding small towns (10 practices) and rural areas (7 practices), thus being representative for most parts of Germany except for large cities. All, but one of the practices were part of the educational network of the university.The 986 patients receiving OAT with phenprocoumon, which is the drug most widely used in Germany for OAT, were identified by searching the GPs’ electronic medical records for the laboratory billing code for INR measurement. The inclusion criteria were age (≥18 years), receiving OAT and willingness to participate in the study. For patients not managing OAT themselves, their caregivers were invited to participate as a substitute but will not be reported here. Exclusion criteria were: inability to give informed consent, management of anticoagulation with self-monitoring (PSM), nursing home residency, insufficient command of German and patients only seen in cross coverage. New oral anticoagulation agents like dabigatran and rivaroxaban had entered the European market in August and December 2011, respectively, and were therefore rarely used during the time of patient recruitment in 2010–11. A total of 577 patients, representing 59% of all potentially eligible patients, were contacted by the practice nurse in order to fix an appointment for INR control. Finally, 345 patients were included in the study. The recruitment is shown in Figure 1.

### Data collection

Demographic data of the participants and patient knowledge about OAT were collected at baseline with a self-developed questionnaire based on models from the literature [14,15]. We chose to use a self-developed questionnaire, because we felt that existing instruments were not comprehensive enough or were not sufficiently adaptable to the specific situation in Germany. The questionnaire evaluated patient knowledge about general information on OAT, and relevant facts regarding nutrition, drug-interactions and other safety precautions such as recognition of complications and informing health care professionals about OAT. Questions were based mainly on relevant national guidelines and expert knowledge [3,10,16]. The questionnaire was piloted previously with 12 patients in a practice, which did not participate in the trial using the think aloud technique [17] and optimized accordingly to ensure comprehensibility.

An English version of the questionnaire is available online [13]. Participants had to fill out the questionnaire themselves under supervision in the practice, without any assistance. Patient names and other confidential information were coded to make it difficult to track individual patients. Informed consent and questionnaires were returned to the study center separately by the practice.

### Statistical analysis

Categorical data were summarized as frequencies and percentages, quantitative variables as medians and interquartile ranges (IQR). For the 13-item knowledge score, all thirteen questions were weighted equally with one point. The total-sum score ranges from 0 to 13, with higher scores indicating greater knowledge.

For questions with multiple yes/no answers the one point awarded to the question in total was divided across all answers. Correct answers received twice the weight of incorrect answers. Indicating “I don’t know” led to 0 points irrespective of which other correct or incorrect answers were ticked. For instance, in question 12 the first four answers were correct, the fifth answer was incorrect, the sixth was “I don’t know”. The four correct answers were weighted by 2/9 points each. The fifth answer which was incorrect carried a weight of 1/9. “I don’t know” carried no weight. For example ticking the first four answers in question 12 gave the maximum score of 1 point. Ticking the first five answers was awarded 8/9 points. Ticking all six answers resulted in no point at all.

To explore factors associated with the score we conducted univariate and multiple regression analyses fitting linear mixed effects models with age, gender, years of education (dichotomized to “less” and “more or equal” than 10 years), fear of bleeding, history of prior OAT related complications, self-rated knowledge as fixed effects, and practice as random effect. The latter accounted for possible correlations between patients from the same practice and thereby adjusted for possible cluster effects. SAS 9.3 (SAS, Cary, NC) was used for statistical analysis. P-values were not adjusted for multiplicity and p-values smaller than 0.05 are referred to as statistically significant.

## Results

### Participants’ characteristics

Of the 345 patients, roughly half (163; 47%) were women. The median age of the participants was 74 years (IQR: 68–78) and 253 participants (73%) had an educational level of less than 10 years of school education (Figure 1). The most common indication for OAT was atrial fibrillation (70%). Patients receiving oral anticoagulants for recurrent thrombosis or pulmonary embolism were younger than those with atrial fibrillation: 75 years (IQR 69–79) versus 72 (IQR 63–78) years. A total of 9% of the participants reported previous complications related to OAT. More than half (54%) of all participants were afraid of complications related to OAT (Table 1). To assess for presence of selection bias we compared age and gender between patients finally enrolled in the study, patients declining to participate and patients who were contacted by the practice. There were no statistically significant differences (results not shown).

**Table 1:** Socio-demographic and clinical characteristics (n = 345)

**Socio-demographic characteristics**	**n (%)**
**Gender**	
Women	163 (47%)
Men	182 (53%)
**Age** (median/IQR***)	74 years (68–78)
Education*	
< 10 years education	253 (73%)
≥ 10 years education	92 (27%)
Indication for oral anticoagulation according to the patient**	
Atrial fibrillation	239 (70%)
Deep vein thrombosis	70 (21%)
Pulmonary embolism	35 (10%)
Artificial heart valve	22 (6%)
Unknown to the patient	16 (5%)
Missing	5 (1%)
**OAT related complication in history**	29 (9%)
**Self-rated knowledge about OAT**	
Excellent	31 (9%)
Good	162 (47%)
Moderate	122 (36%)
Poor	27 (8%)
Missing	3 (1%)
**Afraid of complications**	182 (54%)

* < 10 years corresponds to lowest educational level, more than 10 years to a medium to high educational level.

**Multiple indications for oral anticoagulation therapy (OAT) possible.

*** IQR = interquartile range.

### Patient knowledge

Most participants knew their indication for OAT (95%) and how frequently their coagulation status should be controlled (82%), but in most other areas we observed substantial knowledge gaps (Table 2). A total of 80% of participants did not know which non-prescription analgesic is the safest option for them and 17% indicated they would take non-steroidal anti-inflammatory drugs (NSAIDs). 68% of participants did not know that they had to follow a specific diet. Women had a slightly better knowledge on questions regarding nutrition. Most of the participants were not aware that gastroenteritis, fasting, or non-prescription medicines, could affect the effectiveness of OAT (82%, 86% and 79%, respectively). Many participants were not aware of complications of over- and under-dosing. For example, paresis and melaena were not considered an emergency by 74% and 60% of the participants, respectively (Table 2). Most participants (88%) indicated they would inform health professionals about their OAT before undergoing planned invasive medical procedures. However, 72% of the participants would not inform their pharmacists about OAT.

**Table 2:** Answers to questionnaire assessing knowledge on oral anticoagulation therapy (n = 345)

**#**		**Correct answer**	**Correct n (%)**	Incorrect* n (%)	**Score**
1	Indication for OAT known	Individual	329 (95%)	16 (5%)	1
2	Awareness of risk treated with OAT	Individual	278 (81%)	67 (19%)	1
3	Duration of treatment known	Individual	249 (72%)	96 (28%)	1
4	Frequency of controls known	Individual	278 (81%)	67 (19%)	1
5	Awareness of target INR range	Individual	158 (46%)	187 (54%)	1
6	Need to follow a specific diet				
	Overall		107 (31%)	238 (69%)	1
	• Consuming large amounts of salad and vegetables	No		11 (3%)	0
	• Avoid salad and vegetables	No		57 (17%)	0
	• Regular diet of salad and vegetables	Yes	107 (31%)		1
	• Do not know	No		170 (49%)	0
7	Vitamin K content of some foods				
	Overall				1
	• Cabbage	Yes	245 (71%)	100 (29%)	1/8
	• Potatoes	No	283 (82%)	62 (18%)	1/8
	• Apples	No	284 (82%)	61 (18%)	1/8
	• Green salad	Yes	155 (45%)	190 (55%)	1/8
	• Tomato extract	No	260 (75%)	85 (25%)	1/8
	• Spinach	No	164 (48%)	181 (52%)	1/8
	• Onion	Yes	37 (11%)	308 (89%)	1/8
	• Zucchini	No	268 (78%)	77 (22%)	1/8
8	Management of missed medication dose	n.a.	46 (13%)	299 (87%)	1
9	Awareness that there are no symptoms of underdosing	n.a.	194 (56%)	151 (44%)	1
10	Safest over the counter pain medication				
	Overall		68 (20%)	277 (80%)	1
	• Paracetamol/acetaminophen	Yes	68 (20%)		1
	• Aspirin (acetylsalicylic acid)	No		21 (6%)	
	• Other non-steroidal anti-inflammatory drugs	No		37 (11%)	
	• Do not know	No		219 (63%)	
11	Interaction with OAT				
	Overall				1
	• Regular exercise	No	264 (77%)	81 (23%)	1/8
	• Gastroenteritis	Yes	60 (17%)	285 (83%)	1/8
	• Fever	Yes	16 (5%)	328 (95%)	1/8
	• Coffee	No	327 (95%)	18 (5%)	1/8
	• Ginkgo biloba	Yes	16 (5%)	329(95%)	1/8
	• Non-prescription drugs	Yes	71 (21%)	274 (79%)	1/8
	• Moderate intake of alcohol	No	272 (79%)	73 (21%)	1/8
	• Fasting/weight reduction diet	Yes	48 (14%)	297 (86%)	1/8
12	Recognition of emergency situations				
	Overall				1
	• Painful swelling with or without skin discoloration	Yes	95 (28%)	250 (72%)	1/5
	• Sudden speech disorder	Yes	169 (49%)	176 (51%)	1/5
	• Black stool (melaena)	Yes	133 (39%)	212 (61%)	1/5
	• Arm weakness (also temporarily)	Yes	85 (25%)	260 (75%)	1/5
	• Every cut or injury with bleeding	Yes	224 (65%)	121 (35%)	1/5
13	Important situation to inform others about OAT				
	Overall				1
	• Dental visits	Yes	312 (90%)	33 (10%)	1/5
	• Pharmacist	Yes	93 (27%)	252 (73%)	1/5
	• Before injections	Yes	156 (45%)	189 (55%)	1/5
	• Any new prescription medication	Yes	191 (55%)	154 (45%)	1/5
	• Before invasive medical procedures	Yes	305 (88%)	40 (12%)	1/5
				**Maximum score**	**13**

OAT oral anticoagulation therapy, n.a. non applicable. *non-response was treated as incorrect answer.

### Overall 13-item score and self-rated knowledge

**Figure 2:**
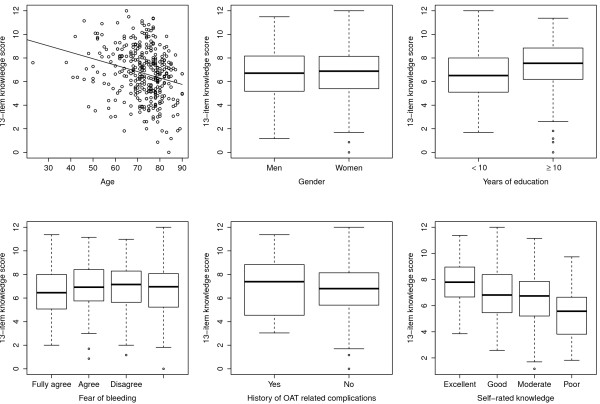
Association of the 13-item knowledge score with age, gender, years of education (dichotomized to smaller, and larger or equal 10 years), fear of bleeding, history of prior OAT related complications and self-rated knowledge.

Participants rated their knowledge about OAT as excellent (9%), good (47%), moderate (36%) and poor (8%) (Table 1). The median overall score was 6.8 (IQR 5.2-8.1). There was a small statistically significant association between self-rated knowledge and age below 65 years towards a higher overall-score in univariate analysis. Patients who rated their knowledge as excellent had a median score of 7.8 (IQR 6.6– 8.9) and those who rated their knowledge as poor of merely 5.6 (IQR 3.8 – 6.6). In univariate analyses higher education and lower age were statistically significant related to higher score, but not gender, history of prior OAT related complications (Figure 2). The observed small differences for age and education remained associated in multivariate analysis (Table 3). Age had a negative coefficient of −0.044 with each year of age. The 21 patients who did not know why they were taking OAT had on average 1.8 points less (p <0.001) on the overall score disregarding this item.There were additionally 22 caregivers participating as substitutes for patients in this study (Figure 1). The data on these substitutes has not been included in the general analysis to avoid bias. Overall they achieved a better median score than patients. But the numbers of caring relatives was too low for meaningful statistical inference.

**Table 3:** Factors associated with the 13-item knowledge score oforal anticoagulation therapy (n = 345)

	**Univariate**		**Multivariate**	
**Factor**	**Effect (SE)**	**p-value**	**Effect (SE)**	**p-value**
Age (in years)	−0.054 (0.1)	<0.01	−0.044 (0.01)	<0.001
Gender (women vs. men)	−0.11 (0.23)	0.64	0.02 (0.23)	0.92
Education				
< 10 years education vs. ≥ 10 years education	−0.68 (0.27)	0.01	−0.74 (0.26)	0.004
Self-rated knowledge				
Good vs poor	2.47 (0.40)	<0.001	2.29 (0.56)	<0.01
Excellent vs poor	1.63 (0.42)		1.44 (0.45)	
Moderate vs poor	1.24 (0.42)		1,06 (0.46)	
history of OAT related complications none vs complications	0.04 (0.41)	0.93	−0.28 (0.39)	0.47
Fear of bleeding				
Fully agree vs disagree entirely	−0.32 (0.33)	0.53	−0.12 (0.32)	0.51
Agree vs disagree entirely	0.09 (0.35)		0.28 (0.34)	
Do not agree vs disagree entirely	0.026 (0.35)		−0.13 (0.32)	

OAT oral anticoagulation therapy.

## Discussion

### Summary of the main results

The results of our survey on patient knowledge show relevant knowledge gaps affecting safe and effective OAT. There was a discrepancy between self-rated knowledge and evaluated actual knowledge. Many participants did not recognize symptoms of emergency situations and had poor awareness of drug interactions, particularly regarding over the counter medication (OTC) and other factors influencing the effects of oral anticoagulants. The overall results are unsatisfying and concerning given that a low level of patient knowledge about OAT is the major cause for bleeding complications [4-6]. Lower levels of education, older age and unawareness about the indication for OAT were associated with lower knowledge about OAT.

### Meaning of the results and comparison with literature

More than half of the patients rated their knowledge on OAT as good or excellent. Despite a correlation between self-evaluation and overall score the actual knowledge was overall low and poor in many safety relevant areas. This confirms findings of many smaller studies [15,18-22].

The majority of patients in our survey knew why they were receiving OAT. Not knowing the indication for OAT was a predictor for poor knowledge. Therefore asking patients why they are taking OAT might be useful to identify patients with an urgent need for instructions. A Spanish study and an Italian study observed that the time spent in the therapeutic range was lower in elderly patients not aware of their indication for OAT [19,20]. This is in line with our finding that older age is associated with a lower number of correct answers. This was also observed by others [21,22]. Although one study found that older age was not associated with an increased risk for bleeding complications [8], others observed a higher incidence of both bleeding and thromboembolic events with advancing age [7]. Cognitive impairment in elderly patients has been associated with inadequate INR control [23].

Only one third of the participants were aware that patients on OAT should follow a regular diet of salad and vegetables to ensure steady vitamin K supply. It has been shown that eating a diet high in vitamin K, reduces the risk of INR measurements in the subtherapeutic range [24]. A significant proportion (11%) was possibly misinformed and assumed that they should avoid vegetables. Avoiding vegetables to minimize variation in vitamin K intake is not compatible with a healthy diet, as recommended by current guidelines [25]. This dietary restriction is frequently perceived as a significant reduction in quality of life by many patients [26].

We observed a low awareness of drugs affecting the pharmacodynamics of OAT, particularly with OTC medication and herbal medicines. Popular herbal medicines like ginkgo biloba extracts and St. John’s wort can affect the risk of bleeding [27,28]. It is known that aspirin and NSAIDs increase bleeding risk, when used in combination with OAT [29]. Most participants did not know which OTC analgesic is safe for patients with OAT and even worse some indicated they would buy aspirin or other OTC NSAIDs. Only 20% were aware that paracetamol (acetaminophen) is considered the safest OTC analgesic for patients on OAT with phenprocoumon [30,31]. This lack of knowledge is particularly worrisome since two thirds of the participants indicated they would not inform pharmacists about concomitant use of OAT when purchasing OTC medication. However, most participants would inform dentists and physicians before undergoing invasive medical procedures.

More than half of the participants did not recognize emergency situations such as symptoms of stroke or bleeding complications as an emergency situation in which they should seek immediate medical attention. Several studies have demonstrated that awareness of symptoms of stroke and recognition of stroke as an emergency in the general populations is low [31]. A group of patients with increased risk of stroke should have higher awareness in order to take appropriate action, if needed.

### Implications for practice

Patients who have already experienced complications related to OAT should be offered instructions or should seek instructions themselves to avoid future complications. However, no higher levels of knowledge were observed in this patient group.

Overall the results of our survey are worrisome and call for structured programs to ensure patient knowledge necessary for safe and effective OAT. It cannot be assumed that one educational intervention is sufficient to maintain necessary knowledge. Although we did not formally assess previous educational activities on OAT, we are aware of the fact that many patients in our sample had been taking OAT for a number of years and had been exposed some form of formal or informal education, regarding their therapy. Therefore periodical reassessment might be necessary. The best strategy for an education program about oral anticoagulation has not been determined yet [10]. An Italian study evaluated the short-term effects of an educational program that did not improve the time spent in the therapeutic range in the short term [32]. In contrast, a French study could show that an education program lowers the complication rate of patients on OAT [33]. A systematic review on the effectiveness of educational interventions to improve the proportion of time spent in the therapeutic range was inconclusive [11]. The included studies were too small to make inferences on complications. Many questions about the best strategy to educate anticoagulated patients remain to be answered. However significant differences in health care systems and the management of OAT will most likely result in different solutions.

### Strength and limitations

To our knowledge this is the largest study, assessing patient knowledge about OAT in a representative sample of primary care patients [11]. Previous studies on patient knowledge were conducted in hospitals or with mostly younger patients [22] often within the frame of patient self-monitoring [9,34]. The results of these studies can therefore not be generalized to the majority of elderly patients [9]. Studies assessing patient knowledge are prone to selection bias. However, we have a high contact and participation rate and comparison of age and gender did not show statistically significant differences of contacted patients and refusing patients compared to participants. We did not inquire about previous education on OAT since there is no generally established education program for OAT in Germany [4]. Although we did not assess the duration of OAT, we believe that the majority of patients included in this study have been taking OAT for several years.

## Conclusions

We demonstrate that patients in the general practice setting have relevant knowledge gaps, potentially affecting safe and effective OAT. GPs should be aware that most patients overestimate their knowledge. There is a need to assess patient knowledge and for effective education program which might need to be repeated periodically. Education programs should focus on drug interactions with non-prescription drugs, dietary advice and recognition of emergency situations. This need for education will persist even when vitamin-K antagonist will be replaced with new drugs.

## Abbreviations

GP: General practitioner; INR: International normalized ratio; IQR: Interquartile range; NSAIDs: Non-steroidal anti-inflammatory drugs; OAT: Oral anticoagulation.

## Competing interests

The authors declare that they have no competing interests. Stefan Vormfelde is employee of Novartis since February 2014, thus after manuscript submission and without influence on any part of the paper.

## Authors’ contributions

Funding for the study was obtained by JFC and SVV. All authors were involved in the design and conduct of the study. The analysis of data was the main responsibility of TF and SS. All authors contributed to manuscript drafting and revision and approved the final manuscript.

## Pre-publication history

The pre-publication history for this paper can be accessed here:

http://www.biomedcentral.com/1471-2296/15/104/prepub
